# Management of Calvarial Osteoradionecrosis After Treatment of Cutaneous Malignancy: A Systematic Review

**DOI:** 10.1002/ohn.1290

**Published:** 2025-05-05

**Authors:** Siyuan Pang, Elizabeth Concannon, Martin Higgins, Katharine Drummond, Peter Gearing, Maxim Devine, Albert Tiong, Anand Ramakrishnan, Carly Fox

**Affiliations:** ^1^ Department of Plastic and Reconstructive Surgery The Royal Melbourne Hospital Parkville Victoria Australia; ^2^ Department of Surgery, The Royal Melbourne Hospital University of Melbourne Parkville Victoria Australia; ^3^ Department of Plastic and Reconstructive Surgery Peter MacCallum Cancer Centre Melbourne Victoria Australia; ^4^ Department of Radiation Oncology Peter MacCallum Cancer Centre Melbourne Victoria Australia; ^5^ Department of Neurosurgery The Royal Melbourne Hospital Parkville Victoria Australia

**Keywords:** complication of radiotherapy, cutaneous malignancy, head and neck cancer, osteoradionecrosis, radiotherapy

## Abstract

**Objective:**

Osteoradionecrosis (ORN) is a recognized complication of radiotherapy. However, calvarial ORN (ORNC) following treatment for cutaneous malignancies is poorly understood. Shedding light on the limited evidence to date, this study aims to summarize existing evidence and highlight the research gaps.

**Data Sources:**

PubMed and Embase.

**Review Methods:**

A systematic search of PubMed and Embase was conducted up to May 2024, using relevant keywords to identify papers on ORNC. Data on the definition, incidence, risk factors, radiotherapy parameters, diagnostic methods, and management strategies were collected.

**Results:**

Twenty articles reporting on 42 ORNC patients were identified, revealing relatively poor data quality. There remains no consensus on a definition of ORNC. Patient‐ and disease‐related risk factors have been inconsistently documented. No conclusion could be reached regarding thresholds for radiation dose delivery, as only seven studies reported the total radiation dose, with a mean of 58.9 Gy. Twenty‐five (60%) cases managed with wound care and antibiotics demonstrated a spectrum of success rates, while 90% of surgically managed ORNC wounds healed at various follow‐up points, ranging from 6 weeks to 9 years.

**Conclusion:**

This study proposes an ORNC‐specific definition and reporting standard through comparison with mandibular ORN. This will help generate valuable data, guiding the development of an ORNC‐specific treatment protocol and clinical decision‐making in managing this debilitating side effect of radiotherapy.

Radiation therapy is commonly used as either definitive or adjuvant treatment for head and neck malignancies, including primary cutaneous scalp cancers. It is associated with a spectrum of immediate and long‐term side effects, which encompasses the risk of developing osteoradionecrosis (ORN).^1^ ORN occurs at an incidence of 3% to 15% following radiation treatment in the head and neck region and is characterized by exposed bone with overlying soft tissue necrosis that fails to heal in 3 months or more in the absence of recurring malignancy.^2^, ^3^, ^4^

Current understanding of ORN is primarily based on studies investigating mandibular ORN (MORN) exclusively. This is evident in the various definitions, classification systems, and management protocols explicit to the mandible.^5^, ^6^ Although the mandible is the most common site, ORN in the head and neck region has the potential to affect any bones within the radiated field, causing varying degrees of morbidity that impact patients' quality of life such as pain, cosmetic and functional issues relating to chronic wound management and infective complications. In comparison to the mandible, there is a paucity of literature concerning ORN occurring at the calvarium (ORNC).

The calvarium, also termed the cranial vault, consists of the paired frontal and parietal bones, part of the temporal bone and the occipital bone.^7^ Along with the overlying scalp, the anatomy and vascular supply to this region pose unique challenges for wound healing due to its convex shape and the relative inelasticity of native scalp tissue.^8^ Radiation as part of cancer treatments can further hinder wound healing, resulting in both early and delayed complications of radiotherapy, for example, wound dehiscence and necrosis.^9^ Without adequate management, ORNC can lead to osteomyelitis and intracranial infections.^10^

This systematic review aims to assess the existing evidence on ORNC, identify research gaps in comparison to the evidence base for MORN, and provide guidance for future research and clinical decision‐making.

## Materials and Methods

### Systematic Review

A systematic electronic database search was performed on May 11, 2024, in accordance with the Preferred Reporting Items for Systematic Reviews and Meta‐analyses (PRISMA) guideline and was registered on the International Platform of Registered Systematic Review and Meta‐analysis Protocols (INPLASY202520117).^11^, ^12^ The search of PubMed and Embase was conducted using a combination of keywords related to osteoradionecrosis, radiation, wound, scalp, and the cranium. The following Medical Subject Headings (MeSH) terms were used: “osteoradionecrosis,” “scalp,” and “head and neck neoplasms.” Search results were filtered to exclude animal and non‐English literature, with no other research limits imposed. Reference lists of all eligible articles and relevant reviews were examined to identify additional studies that were not found in the initial search. Full research strategies can be found in Supplemental Materials S1 and S2, available online.

**Figure 1:**
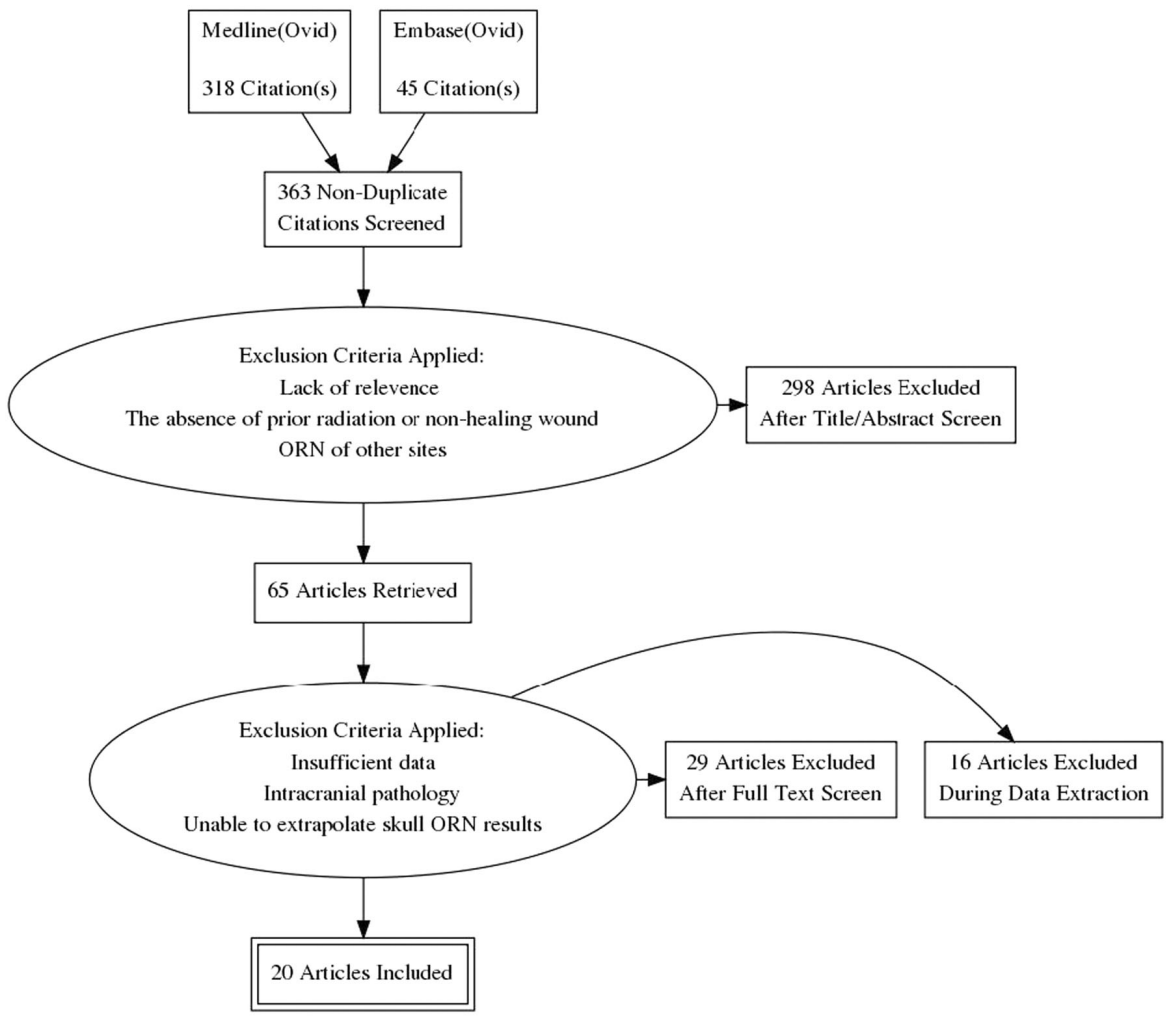
Flow diagram of article selection process. ORN, osteoradionecrosis.

PubMed yielded 318 papers, and Embase provided an additional 45 papers after removing 2 duplicates. Two hundred and ninety‐eight papers were excluded during the title and full‐text screening. Sixty‐five full texts were found, of which 45 papers were further excluded. No additional eligible papers were found during reference screening. Ultimately, 20 papers are included. Figure 1 is the flow diagram of the article selection process.

### Inclusion/Exclusion Criteria for Systematic Review

Studies were included if they addressed a persistent non‐healing scalp wound in the calvarial region following radiation therapy for malignancies. Studies were excluded based on the following predetermined criteria:

1. Lack of relevance: Articles were excluded if they did not discuss or evaluate a non‐healing wound in the setting of prior radiation.
2. ORN of sites other than the calvarium: Articles investigating ORN of other body sites and non‐calvarial head and neck sites such as the skull base, facial skeleton, and occipital‐cervical junction were excluded.
3. ORNC caused by radiotherapy treatment for intracranial pathologies: This review focuses on situations where radiation is specifically targeted at cutaneous scalp tissue alone, to mitigate potential confounding factors, arising from variations in surgical interventions (such as craniotomy) and radiotherapy regimes.
4. Insufficient results or inability to extrapolate results specific to ORNC.

### Data Extraction and Quality Assessment

Three independent reviewers were involved in abstract screening, full‐text review, and data collection. Discrepancies were resolved through group discussion. Data on the definition, incidence, risk factors, radiotherapy parameters, diagnostic methods, and management strategies were collected and formatted into summary tables using Microsoft Word. Quality was assessed using the JBI Appraisal Tool for Case Reports and Case Series (Supplemental Material S3, available online).^13^ Risk of bias and certainty were not assessed using established tools due to the types of studies available; this will be discussed in the Limitations section.

## Results

As shown in Table 1, 42 patients aged between 55 and 93 with ORNC were identified from 20 articles, of which 12 were single‐case case reports.^14^, ^15^, ^16^, ^17^, ^18^, ^19^, ^20^, ^21^, ^22^, ^23^, ^24^ Additional ORNC cases were extracted from three case series^10^, ^25^, ^26^ and four retrospective reviews^27^, ^28^, ^29^, ^30^, ^31^ that primarily focused on MORN or the reconstruction of exposed calvarium irrespective of prior radiation.

**Table 1:** Summary of Literature on Osteoradionecrosis (ORN) of the Calvarium ORN Showing Study Type, Patient Demographics, Comorbidities, Primary Disease, and Treatment

	Country	Year	Study type	Number of patient	Patient age	Patient sex	Patient comorbidities	Primary pathology	Radiation received	Pre‐ORN surgery
Beroukhim and Rotunda^14^	United States	2014	CR	1	76	M	NR	SCC	NR	Excision
Kumbla and Myers^15^	United States	2020	CR	1	75	M	T2DM Hypertension Hyperlipidemia	SCC	NR	Excision
Ghadiri et al^16^	United Kingdom	2021	CR	1	69	M	NR	SCC	54 Gy in 20 fractions	Excision Integra STSG
Momoh et al^17^	United States	2009	CR	1	74	M	NR	SCC Melanoma	NR	Excision
Nguyen et al^18^	United States	2011	CR	1	58	F	NR	BCC	60 Gy; 51 Gy (24 y apart)	Excision
Laman et al^19^	United States	1992	CR	1	69	F	Leukemia Coronary diseases Heart failure Leukopenia	BCC	48 Gy in 16 fractions 45 Gy in 15 fractions (2 mo apart)	Excision
Fernandez^20^	United States	2019	CR	1	87	M	Renal transplantation for PKD	Melanoma	60 Gy in 30 fractions	Excision
Kwee et al^21^	Australia	2012	CR	1	83	M	NR	MM	NR	Excision Local transposition flap STSG
Selber et al^22^	United States	2016	CR	1	55	M	T1DM Kidney and pancreas transplantation	Leiomyosarcoma	NR	Excision Failed local rotation flap
Clegg et al^23^	United States	2020	CR	1	65	F	T2DM COPD Hypertension Tobacco abuse Hyperlipidemia	DLBCL	NR (with chemo)	NIL
Skellett and Levell^32^	United Kingdom	2011	CR	1	70	M	NR	CTCL	90 Gy	Excision Burring RFFF
Siegmund and Rustemeyer^24^	Germany	2019	CR	1	77	F	Arterial hypertonia Hemiplegia from previous stroke	Hemangioma	NR	Excision
Lang et al^25^	United States	2006	CS	1 out of 11 patients	NR	F	NR	SCC	NR	Excision
Snow et al^26^	United States	1994	CS	1 out of 91 patients	NR	NR	NR	SCC	NR	Excision
Lloyd et al^10^	United States	2016	CS	3	NR	NR	NR	SCC BCC	Pt 1: 55 Gy Pt 2: 55 Gy Pt 3: 55 Gy	Excision
Ang et al^27^	Canada	2003	RR	1 out of 21 patients	59	M	NR	SCC	NR	NR
Labow et al^28^	United States	2009	RR	1 out of 37 patients	72	M	NR	SCC	NR	Excision STSG
Afifi et al^29^	United States	2010	RR	3 out of 13 patients	NR	NR	NR	NR	NR	NR
Sandel and Davison^30^	United States	2007	RR	1 out of 14 patients^a^	NR	NR	NR	NR	45 Gy central scalp 45 Gy lateral scalp 18 Gy face	NR
Patel et al^31^	Canada	2020	RR	19	93‐73	M 16 F 3	32% diabetes 26% coronary artery disease 26% pulmonary disease 84% dyslipidemia 100% hypertension 63% former smokers	BCC in n = 1 SCC in n = 13 MM in n = 4 MFH in n = 1	NR	Mixed (1/19 with failed unspecified flap)

Abbreviations: BCC, basal cell carcinoma; COPD, chronic obstructive pulmonary disease; CR, case report; CS, case series; CTCL, cutaneous T‐cell lymphoma; DLBCL, diffuse‐large‐B‐cell lymphoma; MFH, malignant fibrous histiocytoma; MM, multiple myeloma; NR, not recorded; PKD, polycystic kidney disease; RFFF, radial forearm free flap; RR, retrospective review; SCC, squamous cell carcinoma; STSG, split‐thickness skin graft; T1DM, type 1 diabetes; T2DM, type 2 diabetes.

^a^ Three patients with scalp ORN caused by radiotherapy for intracranial pathology were excluded.

SCC was the most common cutaneous malignancy for which adjuvant radiotherapy was indicated, followed by BCC and melanoma. The remaining cases resulted from other malignancies, including cutaneous lymphoma,^32^ sarcoma^22^, ^31^ hemangioma,^24^ and diffuse‐large‐B‐cell lymphoma.^23^ Imaging modalities and tissue biopsy were used to define the extent and rule out active malignancy. Computed tomography (CT) was utilized in seven studies, x‐ray and magnetic resonance imaging (MRI) were used in one study, respectively, and six studies included tissue biopsy to exclude malignancy. The interval from radiotherapy to ORNC onset ranged from 3 months to 7 years. In several case reports, patients only presented once they had developed severe complications due to long‐standing ORNC, including fly larvae infestation^16^ and meningitis with concurrent cerebritis.^18^ Microbiology swabs were not routinely carried out or performed. *Pseudomonas* was cultured from the scalp of an immunosuppressed patient, and *Staphylococcus aureus* was detected in a patient with erosive pustular dermatosis and previous T‐cell lymphoma in the two studies that reported microbiology results.^22^, ^32^

Potential patient‐related risk factors for ORNC have not been established. Six studies reported patient comorbidities at baseline, which included diabetes,^15^, ^31^ hypertension,^15^, ^24^, ^31^ coronary artery disease,^31^ pulmonary disease,^31^ dyslipidemia,^31^ immunosuppression,^19^, ^22^ previous chemotherapy,^20^ and smoking history.^31^ Similarly, radiotherapy parameters were not consistently documented. Seven studies (with 11 patients) recorded the total radiation dose; three reported the number of fractions delivered^16^, ^19^, ^20^; and three reported reirradiation.^18^, ^19^, ^32^ The mean total radiation dose received by the 11 patients with recorded data was 58.9 Gy. A caveat to this is that some of these courses of radiotherapy are likely to be hypofractionated; hypofractionated courses were recorded in two of the three studies with known fractionations.^16^, ^19^

Disease‐related factors, such as primary surgical procedures performed in the affected area, have been well‐documented. Initial malignancies were excised and healed by primary closure in most patients. Three studies documented bone burring to encourage granulation and increase skin graft uptake.^25^, ^31^, ^32^ In cases where reconstruction was required, one report described a local transposition flap with STSG,^21^ whereas the other one utilized a radial forearm free flap (RFFF) with STSG.^32^ The use of the dermal substitute *Integra* ^*TM*^ was reported by Ghadiri et al^16^ and had a favorable outcome until the onset of ORNC approximately 13 years later. Failed flap attempts were documented by two articles, suggesting possible vascular issues at baseline.^22^, ^31^

Conservatively and surgically treated ORNC cases are shown in Tables 2 and 3, respectively. Twenty‐five ORNC cases across five studies were solely managed through regular wound care and antibiotic use. One study recorded the defect size for 19 cases, whereas another documented photos that allowed for size estimation.^14^, ^31^ Beroukhim and Rotunda used a topical beta‐blocker (Timolol) in combination with conventional conservative approaches, and the wound was completely re‐epithelialized in 8 weeks.^14^ One study reported infections in all three patients included in the study, whereas another documented a single case of intracranial infection among 19 conservatively managed ORNC patients, subsequently treated with antibiotics.^10^, ^31^ Skellett and Levell documented a severe ORNC case that was only managed conservatively as the patient repeatedly declined surgery, and for whom a cerebral abscess developed, which eventually led to patient death.^32^

**Table 2:** Summary of Conservatively Managed Calvarial Osteoradionecrosis (ORNC) Cases

	Number of patient	Size of ORNC, cm	ORN diagnostic methods	Onset of ORN from RT	Treatment details	Complication	Outcomes
Beroukhim and Rotunda^14^	1	4 × 5^a^	CT	4 y	▪ Non‐operative debridement ▪ Soap + water daily + petroleum jelly + non‐adherent dressing for 3 y ▪ Subsequent use of 0.5% topical timolol BD to wound for 4 mo	Nil	Healed at 8 wk
Snow et al^26^	1	NR	NR	3 mo^b^	▪ Non‐operative debridement every 3 wk ▪ Soap + water + antibiotic ointment daily + non‐adherent dressing	Failed to heal Wound later grafted	NR
Lloyd et al^10^	3	NR	NR	Min 3 y	▪ Soap + water + petrolatum ointment daily ▪ Debridement of loose necrotic bone PRN	Wound infection (3/3)	Healed
Patel et al^31^	19	2‐25 in diameter	NR	NR	▪ Debridement + antibiotics ▪ Daily or bi‐weekly dressing changes	Intracranial infection (1/19) Recurrent malignancy (1/19) New malignancy (1/19)	Healed
Skellett and Levell^32^	1	NR	X‐Ray Biopsy	NR	▪ Refused surgical treatment ▪ Antiseptics ▪ Topical corticosteroids ▪ Calcipotriol cream ▪ Oral flucloxacillin	Cerebral abscess	Died of disease

Abbreviations: BD, twice a day; CT, computed tomography; NR, not recorded; ORN, osteoradionecrosis; PRN, as required; RT, radiotherapy.

^a^ Estimated using photos.

^b^ Estimated using description and publication date.

**Table 3:** Summary of Surgically Managed Calvarial Osteoradionecrosis (ORNC) Cases

					Treatment details			
	Number of patient	Size of ORNC, cm	ORN diagnostic methods	Onset of ORN from RT	Post‐ORN cranio‐reconstruction	Post‐ORN soft tissue reconstruction	Complication	Outcomes
Kumbla and Myers^15^	1	2.5 × 2	CT MRI	2 y	NR	AV loop Free LD flap 1 wk later	Nil	Healed at 8 wk
Ghadiri et al^16^	1	15 × 15	CT	NR	Cranioplasty (titanium mesh)	LD flap STSG	Nil	Healed at 20 wk
Momoh et al^17^	1	7 x 7	CT biopsy	7 y	Nil	Integra STSG	Nil	NR
Nguyen et al^18^	1	NR	CT biopsy	NR	Duraplasty (avascular TFL and biomet resorbable scaffold)	Local rotational flap STSG	MRSA infection; flap necrosis at 8 wk	Healed at 28 wk after complications treated
Laman et al^19^	1	NR	CT biopsy	4 mo	Nil	HBOT Local rotational flap STSG	Cancer recurrence + small graft slough	Healed at 52 wk
Fernandez^20^	1	7.5 × 8.5	NR	4 y	Nil	Cryopreserved umbilical cord graft	Nil	Healed at 52 wk
Kwee et al^21^	1	10 × 10	Unspecified imaging method	NR	Cranioplasty (titanium mesh + PMMA)	Bilateral free ALT flaps + STSG	Partial flap necrosis + donor site breakdown	NR
Selber et al^22^	1	NR	CT biopsy	6 y	Vascularized composite allotransplant		Nil	Healed at 6 wk
Clegg et al^23^	1	NR	Unspecified imaging method	6 mo	Nil	AV loop Free LD flap 1 wk later STSG	Nil	Healed at 6 wk
Siegmund and Rustemeyer^24^	1	NR	MRI biopsy	NR	Cranioplasty (3D‐printed titanium mesh)	RFFF	Nil	Healed
Lang et al^25^	1	NR	NR	NR	Nil	HBOT Free flap of unspecified type and STSG	Cancer recurrence	Died of disease
Ang et al^27^	1	NR	NR	1 y	Calvarial bone graft	Scapular flap	Nil	Healed at 3 yr
Labow et al^28^	1	NR	NR	NR	Nil	LD flap + STSG	Nil	Healed
Afifi et al^29^	3	NR	NR	NR	Cranioplasty (PMMA + titanium mesh)	ALT flap	Wound dehiscence at 3/6/30 mo, respectively	NR
Sandel and Davison^30^	1	NR	NR	NR	Nil	Free flap of unspecified type	Persistent ORN	NR

Abbreviations: ALT, anterolateral thigh; AV, ateriovenous; CT, computed tomography; HBOT, hyperbaric oxygen treatment; LD, latissimus dorsi; MRI, magnetic resonance imaging; MRSA, methicillin‐resistant staphylococcus aureus; NR, not recorded; ORN, osteoradionecrosis; PMMA, polymethyl methacrylate; RFFF, radial forearm free flap; RT, radiotherapy; STSG, split‐thickness skin graft; TFL, tensor fasciae lata.

The remaining articles detailed various reconstructive approaches, including the use of local rotational flaps,^18^, ^19^ microsurgical free flaps,^16^, ^21^, ^24^, ^25^, ^27^, ^28^, ^29^, ^30^ and free flaps through ateriovenous loop creation.^15^, ^23^ Within this group that underwent surgical management, six out of fifteen studies documented the size of ORNC, ranging from 2.5 × 2 cm^2^ to 15 × 15 cm^2^. Six out of seventeen (35%) surgically managed patients developed minor complications of Clavien‐Dindo class II or below, which include infection, flap necrosis, and wound dehiscence. The recurrence of the primary disease affected two patients, one of whom underwent further reconstruction and was healed at the final follow‐up, and one of whom died of disease. Eleven studies documented the disease outcomes in surgically treated patients at final follow‐up, revealing that 10 out of 11 (90%) patients' ORN wounds had healed at various follow‐up points, ranging from 6 weeks to 9 years. Two studies reported the use of hyperbaric oxygen therapy (HBOT) in combination with surgical reconstruction, but the outcomes were equivocal: Laman et al utilized HBOT in conjunction with a local rotation flap, which was complicated by small areas (<1 cm) of graft slough.^19^ In Lang et al, the free flap (of unspecified type) did not survive despite the use of HBOT.^25^ Synthetic and biosynthetic materials have been used as adjuncts in the reconstruction of the dura, calvarial bones and overlying soft tissue. The use of biodegradable scaffolds,^18^ computer‐designed personalized implants,^24^ dermal matrices,^17^ cryopreserved umbilical cord graft,^20^ and vascularized composite allotransplant^22^ were reported.

## Discussion

Overall, the quality of available data for ORNC is low compared to that for ORN occurring at other sites. This is reflected by the study types available, which are mainly case reports and small case series. Low data quality cannot offer definitive answers to our research questions, we therefore draw on the evidence relating to MORN in an attempt to answer the research questions and highlight the current research gaps relating to ORNC.

### Definition

The general definition of ORN is derived from literature pertaining to MORN specifically.^33^, ^34^ Consensus MORN definitions describe a non‐healing defect in a previously radiated field persisting for at least 3 months in the absence of malignancy.^33^ This definition can be used to drive an ORNC‐specific definition: A post‐radiation defect of calvarial bones and overlying scalp that fails to heal within 3 months in the absence of recurring malignancy. Contrarily, existing classification systems for MORN cannot be applied to ORNC, as they utilize bone necrosis specific to the mandibular region to mark the extent of ORN. Therefore, an ORNC‐specific classification system should be established as evidence accumulates.

### Incidence

ORN incidence is generally difficult to derive due to inconsistencies in the definition, diagnostic criteria, length of follow‐up, loss of follow‐up due to death, and data variability between studies.^35^, ^36^ This is further obscured by advances in radiotherapy techniques, which optimize survival yet have conflicting evidence on ORN incidence.^3^, ^36^ According to Mayland and Sweeny, the incidence of ORN in patients who have received radiation for head and neck cancer ranges between 3% and 15%.^4^ The incidence of ORNC may even be higher because of the tendency to under‐report ORN of smaller size as they often do not require surgical input.

### Risk Factors

MORN risk factors are well‐established relative to those for ORNC. For MORN, tumor‐related risk factors, such as cancer stage and local surgical trauma, and patient‐related risk factors such as smoking, poor local site hygiene, and nutritional and immune status, are known to influence disease occurrence.^35^, ^36^ Existing literature, which only minimally documents risk factors for ORNC, suggests that these factors can be divided into patient comorbidities and disease‐related factors, such as prior radiation dose and previous resections of the scalp or skull. Previous resections and failed reconstructive attempts can aggravate or even initiate a cascade of inflammatory changes that lead to a non‐healing wound that is indistinguishable from true ORNC in irradiated patients. Local surgical trauma is an established risk factor for MORN,^3^, ^6^, ^37^ and it may also increase ORNC risk by loss of integrity of overlying protective scalp soft tissue and underlying skull. Note that only three studies in this review documented cortical bone resection, which was performed to encourage skin graft uptake rather than to resect primary disease. Therefore, the impact of this loss of integrity remains unclear. Future studies should consistently categorize potential risk factors, as the development of ORNC is likely multifactorial.

### Radiotherapy Parameters

Accumulated data regarding MORN have led some authors to speculate on possible dose limits for the mandible. Some studies have revealed that most MORN cases occur after radiotherapy of 60 Gy or more, and for every additional 1 Gy to bone, the risk of MORN increases by 7%.^3^, ^36^, ^38^ There is, however, still mixed evidence regarding the relationship between radiation dose and MORN risk; the safe threshold of radiation treatment can only be derived through the accumulation of detailed radiotherapy data.^39^, ^40^

Most ORNC literature was written by surgeons, and due to this lack of radiation oncology input, most radiation data were unavailable. Seven studies reported the total radiation dose, which is not a comprehensive representation of the effect. No studies recorded radiotherapy planning details such as the use of photon or electron beams, whether treatment used advanced planning techniques or more basic clinical mark‐ups and manual calculations, or any details regarding field sizes, target size, or dosimetry. These factors are important in determining the risk of ORNC because dose at depth (ie, to bone) as well as dose homogeneity and hotspots are related to treatment effect. Future studies on ORNC should consistently report radiotherapy planning details, the total radiation dose (in Gray), the number of fractions, and the volume of irradiated bone to provide a baseline for quantifying the relationship between radiotherapy and ORNC risks.

### Diagnostic Methods

X‐Ray, CT, MRI, and histopathology have been used to diagnose MORN.^41^, ^42^, ^43^ On X‐ray, early MORN presents with a reduction in bone density, whereas disease of later stages presents with expansive or fused lamellar low bone destruction, sequestrum formation, or even pathological fracture.^41^, ^42^, ^43^ CT may show similar findings with large lytic areas and adjacent soft tissue swelling or infection.^41^, ^42^, ^43^ MRI can show marrow alteration in early stages, cortical erosions, and surrounding soft tissue damage.^41^, ^42^ Histopathology often shows an absence of osteocytes and non‐vital bone tissues that are replaced by acellular fibrotic tissues.^41^, ^44^ Swabbing for microbiology is not typically a standard diagnostic tool for MORN, as it is primarily a radiation‐induced condition rather than an infectious one. However, secondary infections caused by *S. aureus*, *Pseudomonas aeruginosa*, and other oral flora bacteria can occur.^45^

Radiological and histopathological features specific to ORNC have not been described in the literature. Eleven studies in this review used imaging and histopathology to confirm diagnosis and assess severity, but no study recorded the specific ORNC findings identified. In our experience, radiologic changes in very early ORNC may be difficult to detect. As the pathology progresses, erosion of the outer table and inner table becomes evident, along with marrow changes. These changes must be differentiated from malignancy. To determine the extent of ORNC and define resection margins for surgical treatment, radiologically detected bone changes must be evaluated alongside prior radiation fields, clinically visible effects (eg, wounds, alopecia, and dermal discoloration), CT findings, and intraoperative bone appearance. Currently, we are unable to determine from the literature the ideal combination of these modalities to ensure complete treatment of the affected bone.

Microbiology results have only been reported in two patients with varying degrees of immunosuppression.^22^, ^32^ More data are needed to assess the role of microbiological swabs in identifying organisms that may predispose individuals to developing ORNC, contribute to secondary infections, or help evaluate the use of perioperative antibiotics as part of conservative management, especially in those who are immunocompromised.

### Management Strategies

#### MORN

Conservative management, including antibiotics with regular wound dressing, is often used to treat early or mild disease and has a disease resolution rate of 0% to 44%.^46^, ^47^, ^48^ HBOT has shown controversial effectiveness, with numerous studies both supporting and opposing its use.^49^, ^50^, ^51^ A combination of pentoxifylline, tocopherol, and clodronate (together referred to as PENTOCLO) has shown efficacy in treating mild MORN.^41^, ^46^ Surgical interventions have remained a cornerstone for MORN treatment.^46^ X‐Ray or CT can guide the resection of MORN, which is usually planned with a 1‐cm margin beyond the radiographic changes or until healthy‐appearing bone with fresh bleeding is reached.^6^, ^41^, ^52^ Depending on disease severity, debridement, resection, or complex reconstruction using local or free flaps is required. The exact surgical treatments employed depend on a magnitude of factors, including the area and depth of MORN, quality of surrounding soft tissue, and concurrent facial or intraoral defects that also require reconstruction.^46^

#### ORNC

Currently, for ORNC, treatment decisions rely on the case‐by‐case consideration of factors including disease severity, depth of involvement, and patient comorbidities and preferences. No classification system or treatment protocol specific to ORNC exists at the time of writing.

According to the systematic review by Patel et al, conservative measures can maintain an acceptable quality of life for patients with ORNC.^31^ Future research should quantify the likelihood of developing minor and major complications in conservatively managed patients, so it can be weighed against the risk of surgery, especially for patients with early‐stage disease or high anesthetic risks. In addition, the development of a standardized wound care routine would help minimize the cost associated with long‐term wound dressing and improve postoperative outcomes. Beta‐blockers can speed up cell migration by acting on the adrenergic receptors of endothelial cells, neutrophils, and macrophages.^53^ Their effectiveness has been shown in burn management by reducing recovery time and skin graft requirement.^14^, ^53^ Although Beroukhim and Rotunda showed that topical timolol promotes healing, whether it should be incorporated into the management routine of ORNC awaits further evidence.^14^

Medical treatments, such as PENTOCLO, and HBOT have not been widely used for ORNC. HBOT was reported in two patients with ORNC, but its effects were difficult to judge as both cases involved other surgical measures and were complicated by cancer recurrence.^19^, ^25^ There is limited evidence supporting the use of HBOT in treating ORNC, and a comprehensive review of the literature on HBOT in treating MORN is beyond the scope of this study.

Surgical management remains the mainstay treatment option for ORNC due to limited evidence investigating the role of conservative management. Debridement and resection to healthy margins of skin and bone are required for most patients, but it is not always possible or safe, particularly due to adherent dura over major venous sinuses. Free flaps are preferred over local flaps for ORNC reconstruction, with fasciocutaneous and muscle‐only free flaps such as the anterolateral thigh (ALT) and latissimus dorsi (LD) flap, which can allow resurfacing of large surface areas of the scalp, being frequently reported.^15^, ^16^, ^21^, ^23^, ^28^, ^29^ For these previously irradiated patients, depending on the size of their ORNC defect, local donor tissue of adequate size may not be available. Microvascular free flaps can bring in reliable vasculature and provide sufficient soft tissue coverage. Using LD flaps to reconstruct the scalp has several advantages. In addition to being large and pliable, it is of similar thickness to normal scalp tissue once spread out over a large defect with potential shrinkage over time, making reconstructive contouring easier and providing satisfactory cosmesis. It also has a good pedicle length and vessel size and is relatively radio‐resistant.^54^ However, with time, atrophy of the muscle may lead to exposure of underlying cranioplasty material or incompletely resected ORNC. The ideal flap for coverage of these complex defects is yet to be determined.

For severe ORNC where there is destruction of the outer table into cancellous bone or even inner table, complex cranial vault reconstruction with neurosurgical input is often required. Dural breach is particularly serious due to the risk of intracranial infection. Where possible, surgical management should maintain or restore dural integrity. The need for reconstruction of the bony defect depends on the size, position, cosmetic implications, and patient health and preferences. Cranioplasty should mainly be considered for large scalp defects or those that would notably affect appearance if bone reconstruction is not performed.^55^ Commonly used materials for cranial vault reconstruction include titanium mesh, acrylic, and polyetheretherketone (PEEK), which may or may not be custom 3D‐printed. Previous research has identified that prior radiation often leads to worse cranioplasty outcomes and is associated with an increased infection rate in titanium mesh cranioplasty, but not in PEEK cranioplasty.^56^, ^57^ However, the majority of the literature on this topic addresses cranioplasty in trauma patients or those with intracranial or unspecified tumors, whereas factors such as the type of primary tumor, radiation dose, the size of the soft tissue defect, and the associated methods of soft tissue reconstruction are often neglected in reporting.^58^ The ideal cranioplasty material in ORNC patients who face challenges related not only to radiation and healing but poor soft tissue envelopes due to previous surgery, is yet to be determined. Novel interventions, such as the use of synthetic materials and autologous transplants, require more level I data to demonstrate their medical and financial practicality.

#### Summary

ORNC can be defined as any post‐radiation defects of calvarial bones and overlying scalp that fail to heal within 3 months in the absence of recurring malignancy. Table 4 presents the proposed reporting standard for future ORNC studies, aiming to collect more valuable data that would guide the development of an ORNC‐specific treatment protocol and, consequently, inform treatment decisions.

**Table 4:** Summary of Suggested Data Points for Future Calvarial Osteoradionecrosis (ORNC) Studies

Suggested data points for future ORNC studies	
Incidence	▪ Percentage of ORNC among patients who have received radiotherapy for head and neck cancer.
	▪ Duration between radiation and disease onset.
Risk factors	▪ Patient‐related factors: conditions affecting wound healing, immunosuppression, vasculopathy, and smoking.
	▪ Disease‐related factors: size and location of primary lesions, extent of bone burring and resection, and method of closure and reconstruction.
	▪ Radiation planning details: type of beams (photon/electron), use of advanced planning techniques versus basic clinical methods, and specifics on field sizes, target size, or dosimetry.
	▪ Radiation treatment details: total dose (Gray), number of fractions, and volume of irradiated bone.
Diagnostics	▪ Imaging findings.
	▪ Correlation of imaging findings to previously treated radiation field and extent of disease.
	▪ Histopathologic findings.
	▪ Microbiological findings.
Management	▪ Methods of conservative management.
	▪ Success and complication (both minor and major) rates of conservative management and the criteria for considering surgery in those who fail to heal.
	▪ Extent of bone resection (currently affected area versus entire radiation field).
	▪ Role of perioperative antibiotics.
	▪ Muscle versus fasciocutaneous free flaps.
	▪ Ideal cranioplasty material.
	▪ Success and complication rates of surgical management.

## Limitation of Study

This study is currently the only comprehensive systematic review on both conservatively and surgically managed ORNC cases. However, certain limitations need to be acknowledged. The quality of this review is limited by the quality of the primary studies, which are primarily case reports and case series with small sample sizes. As a result, the primary data are subject to high degrees of selection and reporting bias. Consequently, the risk of bias and certainty could not be assessed using established tools, such as the Cochrane Risk of Bias tool, which is designed for randomized controlled trials. Secondly, this review excluded ORNC cases caused by intracranial pathologies, including astrocytoma, glioblastoma, meningioma, oligodendroglioma, and neuroblastoma.^59^, ^60^, ^61^, ^62^, ^63^, ^64^ The impact of radiation on intracranial pathologies on the development of ORNC can be confounded by baseline surgery (such as craniotomy) and radiation techniques. This patient group should be analyzed separately as they may skew the dose constraint above which ORNC is more likely to develop following radiation therapy for extracranial malignancies.

## Conclusion

Compared to MORN, which has been comprehensively researched, more site‐specific data are required to investigate ORN occurring at the calvarium. We have formulated an ORNC‐specific definition to establish a foundation for gathering more site‐specific data. Research gaps and the ideal ORNC case reporting standard in terms of its incidence, risk factors, diagnosis, and management were highlighted. Collaboration between surgical and radiation oncology departments is crucial to establish the incidence of ORNC and its associated risk factors. With the accumulation of more comprehensive and consistent data, an ORNC‐specific treatment protocol based on the size and extent of the ORNC can be developed to guide surgical management and decision‐making.

## Author Contributions

**Siyuan Pang**, design, data collection, analysis, drafting/editing, presentation; **Elizabeth Concannon**, design, data collection, drafting/editing; **Martin Higgins**, design, data curation, editing; **Katharine Drummond**, writing/editing, neurosurgical advice; **Peter Gearing**, design, data collection, editing; **Maxim Devine**, design, editing; **Albert Tiong**, writing/editing, surgical oncology advice; **Anand Ramakrishnan**, supervision, editing, plastic surgical advice; **Carly Fox**, supervision, writing/editing, plastic surgical advice, presentation.

## Disclosures

### Competing interests

The authors declare no conflict of interest.

### Funding source

No authors have received any funding or support.

## Supporting information

Supplementary Material 1: Full research strategy (PubMed).

Supplementary Material 2: Full research strategy (Embase).

Supplementary Material 3: JBI appraisal of included studies.
