# Supplementary figures and images for: Management of Calvarial Osteoradionecrosis After Treatment of Cutaneous Malignancy: A Systematic Review
Source: Otolaryngol Head Neck Surg. 2025 May 5;173(3):552–65. doi: 10.1002/ohn.1290 (PMC12379840; doi:10.1002/ohn.1290)

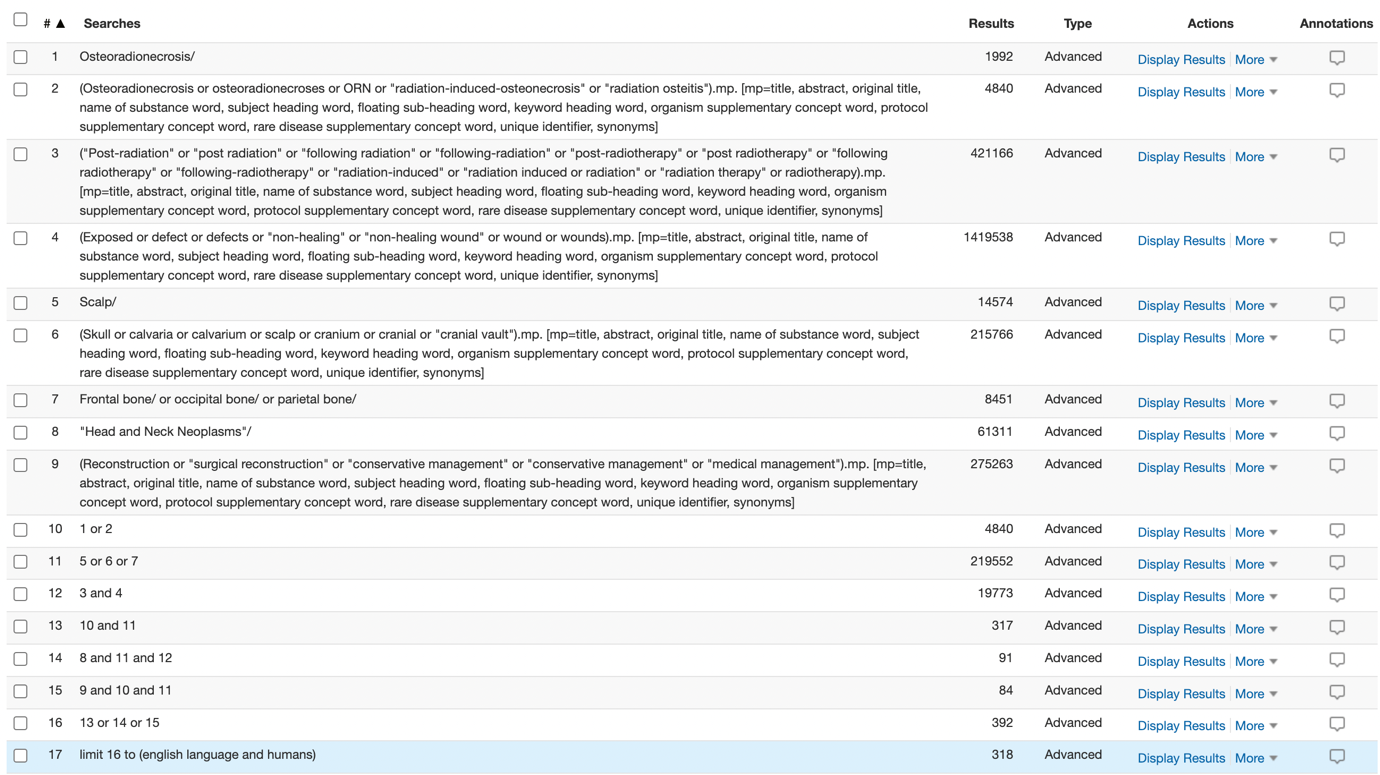


Supplementary Material 1- Full Research Strategy(PubMed)

Supplement: Supplementary file 1 — Supplementary Material 1: Full research strategy (PubMed). [file OHN-173-552-s002.docx]

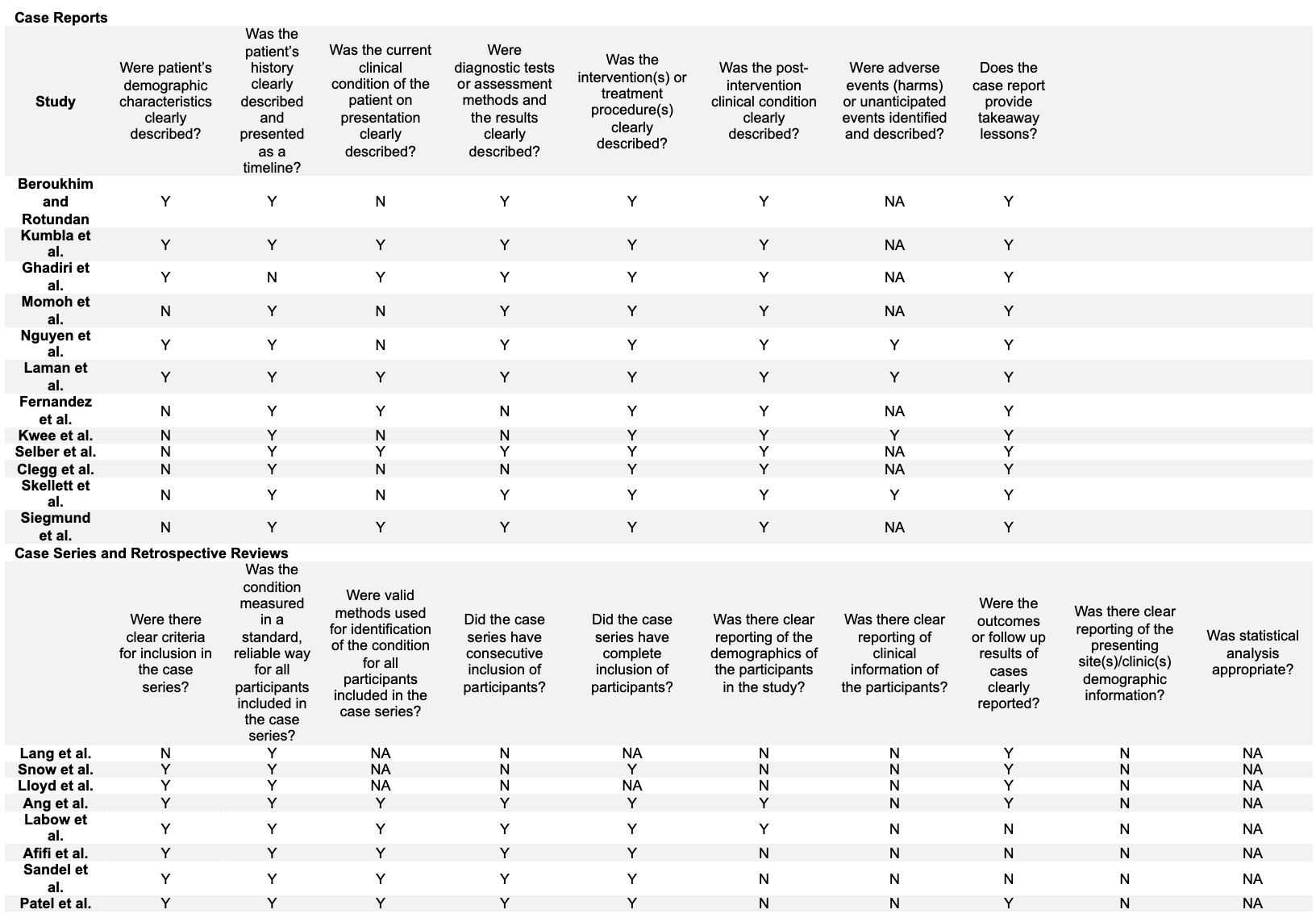


Supplementary Material 3-JBI Appraisal of Included Studies *Y:yes, N:No, NA: Not Applicable

Supplement: Supplementary file 3 — Supplementary Material 3: JBI appraisal of included studies. [file OHN-173-552-s003.docx]
