# Supplementary material for: Management of Calvarial Osteoradionecrosis After Treatment of Cutaneous Malignancy: A Systematic Review
Source: Otolaryngol Head Neck Surg. 2025 May 5;173(3):552–65. doi: 10.1002/ohn.1290 (PMC12379840; doi:10.1002/ohn.1290)
Supplement: Supplementary file 2 — Supplementary Material 2: Full research strategy (Embase). [file OHN-173-552-s001.docx]

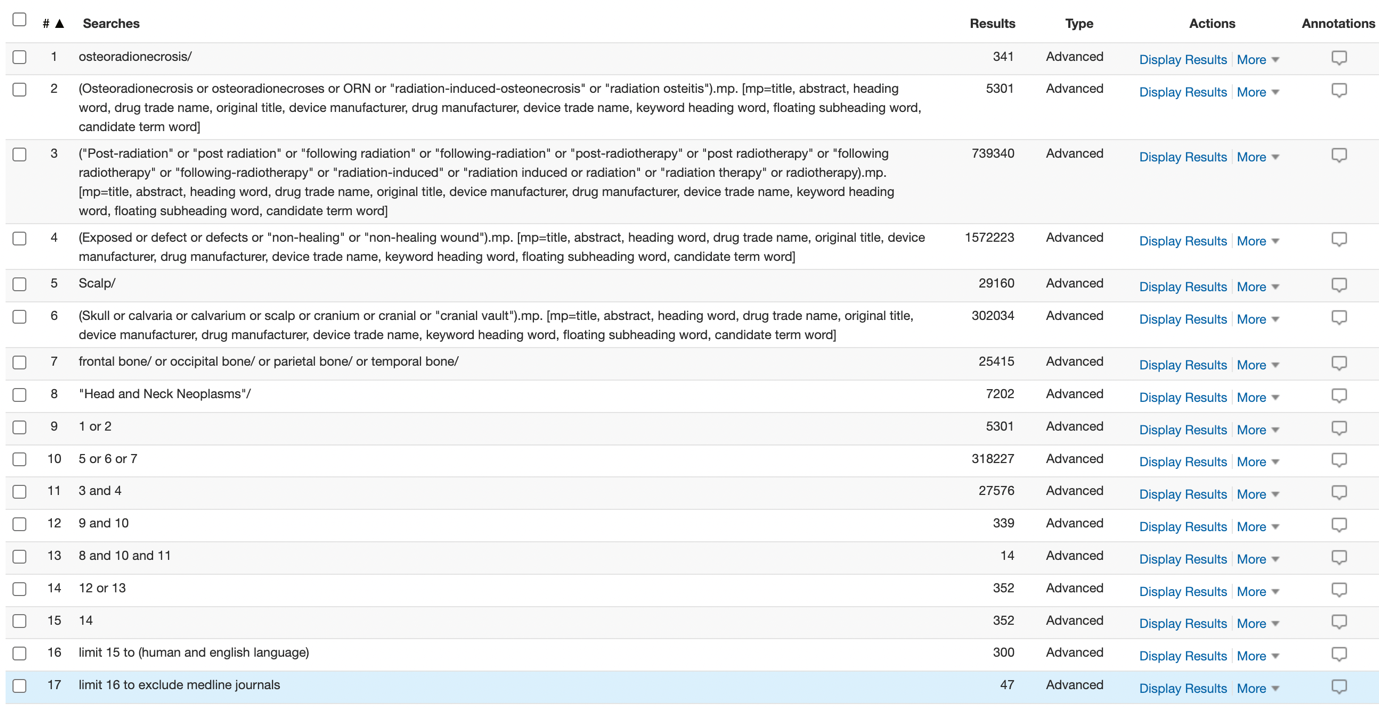


Supplementary Material 2- Full Research Strategy(Embase)*Two additional duplicates were excluded manually.
